# Individual differences in emotion regulation moderate the associations between empathy and affective distress

**DOI:** 10.1007/s11031-018-9684-4

**Published:** 2018-03-14

**Authors:** Philip A. Powell

**Affiliations:** 1 Department of Economics, Institute for Economic Analysis of Decision-making (InstEAD), University of Sheffield, 9 Mappin Street, South Yorkshire, S1 4DT UK; 2 Present Address: School of Health and Related Research (ScHARR), University of Sheffield, South Yorkshire, S1 4DA UK

**Keywords:** Anxiety, Depression, Emotion regulation, Empathy, Stress

## Abstract

Individual differences in empathy can have positive and negative psychological outcomes. Yet, individual differences in the processing and regulation of empathy-induced emotion have not been fully explored within this dynamic. This study was designed to explore whether individual differences in emotion regulation strategies moderated the effects of empathy on common forms of affective distress. Eight hundred and forty four participants completed survey measures of trait empathy, emotion regulation strategies, and symptoms of depression, anxiety, and stress. Affective empathy typically predicted greater affective distress, but the effects on depression and anxiety were offset when people were effective at reappraising their emotions. Cognitive empathy predicted lower distress on average, but this beneficial effect on anxiety and stress was absent in those who typically suppressed their emotions. Finally, suppression unexpectedly reduced the depression and stress reported for people high in affective empathy. Individual differences in emotion regulation are an important moderator between empathy and psychological health, and thus a useful target for intervention.

**Electronic supplementary material:**

The online version of this article (10.1007/s11031-018-9684-4) contains supplementary material, which is available to authorized users.

## Introduction

The capacity to empathise is fundamental to us as social animals. Understanding the anger of a fellow commuter over whom we have just spilled our morning coffee acts as a signal for reparation; feeling and mimicking the joy of a successful peer reinforces social bonds; sharing in the sorrow of others less fortunate contributes to virtues of altruism, justice, and fairness. The capacity for empathy is often viewed as a positive trait, leading to desirable outcomes, including prosocial behaviour and compassion (Singer and Klimecki 2014). The fuller consequences of empathy, however, are multifaceted, and heightened levels of empathy can also have negative ramifications. The ability to read other people’s emotions may underlie narcissistic exploitation (Wai and Tiliopoulos 2012), for example, while empathic distress, via emotion contagion, has been associated with greater depression and reduced psychological wellbeing (Schreiter et al. 2013).

As a primary part of the emotion-generative process, it is unlikely that empathy impacts people’s affective wellbeing in and of itself, but could be hypothesised to have an interactive relationship with the subsequent processing and regulation of emotion (Eisenberg 2000). Different types of emotion regulation strategy have been shown to have positive and negative relationships with psychological wellbeing (Gross and Muñoz 1995). The ability to cognitively reappraise one’s affective states, for example, is often linked to positive psychological outcomes, while the suppression of emotion is frequently associated with negative consequences (John and Gross 2004). Less is known, however, about how individual differences in the use of emotion regulation techniques may potentiate or compensate for underlying differences in dispositional empathy when predicting psychological outcomes (see Eisenberg 2000). In this paper, a theoretical premise is explored that individual differences in emotion regulation may moderate, by enhancing or offsetting, the observed effects of dispositional empathy on symptoms of affective distress.

### Empathy

Definitions of empathy vary, but there is near universal consensus for individual differences in two separable components: a “cognitive” aspect, or the ability to recognise, understand, and react appropriately to others’ emotional states, and an “affective” aspect, or the ability to feel and share others’ emotions (Reniers et al. 2011). These two facets of empathy have been differentially linked to people’s personalities and interpersonal behaviour (e.g., Wai and Tiliopoulos 2012). A recent study measuring affect sharing and the cognitive understanding of others’ mental states simultaneously provided evidence for the constructs’ behavioural and neural independence (Kanske et al. 2016). Moreover, in their theoretical model of empathy, Bird and Viding (2014) sketched a role for theory of mind that is distinct from affect sharing, noting that the former is not necessary for, but may contribute to, the latter.

Prior to considering the evidence on empathy and affective distress, it is important to emphasise the positive social and emotional benefits of empathy for individuals and their relationships. Daniel Batson’s longstanding “empathy-altruism” hypothesis (Batson 2012) posits that empathic concern for others produces the altruistic motivation that underlies subsequent prosocial behaviour. This hypothesis has been supported by over three decades of empirical work (e.g., Batson et al. 1981, 2003). In her review of the field, Eisenberg (2000) notes the capacity for empathic responding to produce either sympathy or personal distress, and details a number of studies demonstrating a relationship between empathy/sympathy and prosocial behaviour in children and adults. Hoffman (2008) argues for the role of empathic distress in motivating helping behaviour, providing it does not become so aversive as to represent a state of “empathic overarousal”. Finally, a recent fMRI study by Lockwood et al. (2016) demonstrated that people higher in trait empathy learned more quickly in a task involving the prosocial rewarding of others and had more selective responses in a region of the brain involved in prosocial learning (the subgenual anterior cingulate corex/basal forebrain). This evidence and the potential clinical correlates of low levels of cognitive (i.e. autistic spectrum disorder) and affective (i.e. psychopathy) empathy (Baron-Cohen 2011) illustrate its important role for normative socio-emotional functioning. Nonetheless, additional evidence also points towards a potential “dark side” of having an empathic capacity, in terms of its relationship with affective distress.

### Empathy and affective distress

Evidence on the association between trait cognitive empathy and affective distress is mixed (e.g., Schreiter et al. 2013). Most work has focused on depression, with much less on other common experiences, such as anxiety and stress. While some studies have noted a significant negative relationship between cognitive empathy and depression (e.g., Cusi et al. 2011), others have found a positive, or no, link (e.g., Dinsdale et al. 2016). In a review, Schreiter et al. (2013) concluded that depression was more strongly related to reduced cognitive empathy than the reverse. Further work has shown that people who are socially anxious may be less accurate at theory-of-mind-related tasks than those with depression or healthy controls (Washburn et al. 2016), but opposing findings have been published (Tibi-Elhanany and Shamay-Tsoory 2011). Park et al. (2015) reported a negative correlation between a cognitive measure of physician empathy and perceived stress. While the literature has inconsistencies, on balance cognitive empathy appears positive for psychological health.

Despite its prosocial benefit, the evidence for heightened affective empathy and its link to affective distress points to a more negative conclusion. Schreiter et al. (2013) identified a positive association between affective empathic stress (i.e., emotion contagion and shared pain) and depressive symptoms. Kahn et al. (2017) reported a significant positive correlation between symptoms of anxiety and affective empathy in young people, while Kaźmierczak et al. (2013) found significant positive correlations between emotional empathy and gender role stress. Thus, while affective empathy appears to be related to increased psychological distress on average, some research has challenged this. For example, in their review, Schreiter et al. (2013) noted the absence of an association between affective empathic concern and depressive symptoms (see Cusi et al. 2011).

One potential explanation for the abovementioned inconsistencies is that the relationship between empathy and affective distress is not linear. A recent study found a significant quadratic, but not linear, relationship between perspective-taking and depressive symptoms (Tully et al. 2016), where very low and high levels of perspective-taking were associated with increased depression. Another potential explanation is that the association between empathy and distress depends on a third variable, such as how people process, or regulate empathy-induced emotion. Tully et al. (2016) showed that a composite of “emotion dysregulation” traits (ruminative brooding, ruminative pondering, and guilt) moderated a quadratic—not linear—effect of empathic concern on depression. This work, however, was limited by the size and composition of its sample, the analysis of perspective-taking and empathic concern in separate models, and the failure to explore the effects of empathy and emotion dysregulation on affective outcomes beyond depression.

### Emotion regulation and affective distress

Emotional experience is the key product of empathy, and has been shown to be a temporal antecedent, as well as a consequence, of affective distress (e.g., Powell et al. 2013). How people respond to empathy-induced emotion is thus a key puzzle piece in understanding the temporal unfolding of empathy and distress. Emotion regulation has been studied widely in relation to psychological health (Gross and Muñoz 1995), and is likely to play a crucial role in determining whether empathy-induced emotion has subsequent positive or negative outcomes (Eisenberg 2000; Hoffman 2008). Two commonly researched emotion regulation strategies include individual differences in “cognitive reappraisal”, or reimagining stimuli in a way that alters its affective impact, and differences in “expressive suppression”, or actively inhibiting emotional expression (Gross and John 2003).

Evidence suggests that a capacity to reappraise emotions is positive for psychological health (John and Gross 2004). Martin and Dahlen (2005) demonstrated negative associations between cognitive reappraisal and depression, anxiety, and stress. On the other hand, suppressing emotional expression has been shown to negatively affect people’s wellbeing (John and Gross 2004). Moore et al. (2008) noted significant associations between expressive suppression and PTSD, anxiety, and stress symptoms in those exposed to trauma. Haga et al. (2009) reported significant links between suppression and reduced life satisfaction and increased depressed mood.

Accordingly, if emotion regulation is as important to psychological adjustment as the evidence suggests, and empathy is an emotion-generative process, it would follow that individual differences in regulatory approaches should act as an important moderator between empathic responses and affective distress (Eisenberg 2000). Such knowledge may be important in further understanding the mechanisms linking empathy to common forms of distress, and in informing interventions designed to disrupt the link between the two. No evidence, however, has surfaced to underpin this theoretical model. The closest piece of work to date, which found a moderating role for proxies of “emotion dysregulation” between empathic concern and depressive symptoms, found no significant moderating role for the emotion regulation variables in a smaller sample of psychology students (Tully et al. 2016).

### Empathy, regulation, and distress: the present study

The current study was designed to test the theoretical model that the link between individual differences in the capacity for empathy and symptoms of affective distress may be moderated by common emotion regulation mechanisms. It was designed to do so using a large sample and modern linear and quadratic regression models.

Based on prior literature (e.g., Schreiter et al. 2013; Tully et al. 2016), the following predictions were made regarding empathy and affective distress:

1. Cognitive empathy would have a negative linear association with measures of affective distress; and
2. Affective empathy would have a positive linear association with measures of affective distress.

Based on the significant quadratic effect of perspective-taking on depression observed by Tully et al. (2016), the following further prediction was made:

(3) Measures of empathy would have a quadratic association with measures of affective distress (i.e., there may be a normative or optimal level of empathy associated with less affective distress, which was neither too low nor too high).

Based on previous findings regarding regulation and distress (e.g., John and Gross 2004; Martin and Dahlen 2005), the following predictions were made:

(4) Reappraisal would have a negative association with affective distress; and
(5) Suppression would have a positive association with affective distress.

Finally, based on the theoretical arguments exposited above, the following predictions were made about the interactive effect of empathy and emotion regulation on distress:

(6) Reappraisal would positively moderate the effects of empathy on affective distress, either by enhancing the positive effects of cognitive empathy and/or negating the negative effects of affective empathy; and
(7) Suppression would negatively moderate the effects of empathy on affective distress, either negating the positive effects of cognitive empathy and/or potentiating the negative effects of affective empathy.

Additional exploratory analyses tested whether the effects of empathy, emotion regulation, and their products differed significantly across three different domains of affective distress.

## Method

### Participants

Eight hundred and forty-four student volunteers (597 women) at the host university took part in this study. Participants’ ages ranged from 18 to 66 years, with a mean of 22.27 years (*SD* = 5.05). The majority were undergraduates (*n* = 555), single (*n* = 451), and UK nationals (*n* = 528).

### Measures

#### Demographics

Participants answered background questions on their gender (0 = male, 1 = female), age, student status (0 = undergraduate, 1 = postgraduate), nationality (0 = UK national, 1 = international), and relationship status (0 = not in an interpersonal relationship, 1 = in an interpersonal relationship).

#### Empathy

Individual differences in empathy were measured with the Questionnaire of Cognitive and Affective Empathy (QCAE; Reniers et al. 2011). For each of 31 items, participants rate their agreement on a 4-point Likert scale (1 = strongly disagree, 4 = strongly agree). Nineteen items measure cognitive empathy (with scores from 19 to 76) and 12 items assess affective empathy (with scores from 12 to 48), with higher scores indicating a higher propensity to empathise. The QCAE has displayed good internal reliability and validity in non-clinical samples (e.g. Reniers et al. 2011), with Cronbach’s alphas of α = 0.87 for the cognitive subscale, and α = 0.88 for the affective subscale (Lockwood et al. 2014). The measure has also been shown to be temporally stable, with high test re-test reliability (*r* = .84) over an average duration of 3 weeks (Powell and Roberts 2017). The cognitive and affective empathy subscales have been shown to correlate moderately in prior work (*r* = .31; Reniers et al. 2011). In this sample, Cronbach’s alphas for the cognitive, α = 0.89, and affective, α = 0.78, subscales were good.

#### Emotion regulation

Differences in emotion regulation strategies were measured with the Emotion Regulation Questionnaire (ERQ; Gross and John 2003). For each of 10 items, participants indicate their agreement on a 7-point Likert scale (1 = strongly disagree, 7 = strongly agree). Six items measure cognitive reappraisal (with scores from 6 to 42) and four items measure expressive suppression (with scores from 4 to 28). Higher scores indicate greater levels of the constructs. The ERQ has been shown to have good construct validity and internal consistency in student samples, with average Cronbach’s alphas of α = 0.79 for the reappraisal subscale, and α = 0.73 for the suppression subscale (Gross and John 2003). The two subscales have been shown to be independent, with an average correlation coefficient close to zero (*r* = − .01, Gross and John 2003). The instrument has also been shown to have good test re-test reliability (*r* = .69) over a duration of 3 months (Gross and John 2003). In the present study, the reappraisal, α = 0.86, and suppression, α = 0.77, subscales demonstrated a good level of internal reliability.

#### Affective distress

Current symptoms of affective distress were measured using the Depression, Anxiety and Stress Scales-21 (DASS-21; Lovibond and Lovibond 1993). For each of 21 items, participants rate how much the statement has applied to them over the past week on a 4-point Likert scale (0 = did not apply to me at all, 3 = applied most of the time). Seven items assess depression, anxiety, and stress. Scores for each subscale range from 0 to 21, and higher scores represent greater symptoms. The DASS has excellent validity and reliability in non-clinical samples (Lovibond and Lovibond 1993). Inter-correlations between the DASS subscales have been shown to be moderate to high (*r* = .54 for depression and anxiety; *r* = .56 for depression and stress; and *r* = .65 for anxiety and stress; Lovibond and Lovibond 1995). The subscales of the DASS-21 appear to have a good standard of internal consistency with Cronbach’s alphas of α = 0.88 for the depression subscale, α = 0.82 for the anxiety subscale, and α = 0.90 for the stress subscale (Henry and Crawford 2005). As a state measure, the DASS-21 has moderate test re-test reliability (*rs* = .46 for the depression subscale over a duration of 12 months; Powell et al. 2013). In the current sample, the depression, α = 0.89, anxiety, α = 0.82, and stress, α = 0.84, subscales displayed good Cronbach’s alphas.

### Procedure

Institutional ethical approval was acquired prior to data collection, and the research was conducted in a manner consistent with the British Psychological Society’s Code of Human Research Ethics. Informed consent was obtained as a prerequisite to accessing the study measures. As part of the sign-up process for a broader study, participants were invited through the author’s host institution’s emailing lists to take part in an online survey on emotion hosted on Qualtrics (http://www.qualtrics.com). Participants completed the demographic questions, QCAE, ERQ, and DASS-21 in a randomised order.

### Data analysis

The distributions of the outcome variables were not Gaussian, but had densities and levels of skewness and kurtosis consistent with a beta distribution (Cullen and Frey 1999; Delignette-Muller and Dutang 2015; see Figs. S1 and S2 in the Supplementary Material). Accordingly, non-parametric Spearman’s rho correlations and beta regressions were used to model the data (see Smithson and Verkuilen 2006). These model the response variable as a proportion of the total score possible bounded between (0, 1), and showed a superior fit to both linear regressions on the original outcome variables, or linear regressions on (log, square-root, and reciprocal) transformed outcome variables that failed to fully correct levels of skewness and kurtosis (the beta models had a considerably lower AIC, see Table S1 in the Supplementary Material). The advantages of beta regression over standard Gaussian models, when outcome variables deviate from normal, are noted in Smithson and Verkuilen (2006). To fit the beta models, the formula cited in Smithson and Verkuilen (2006) was used to convert [0, 1] to (0, 1) proportional data.

Separate regression models are presented for the three primary outcomes (depression, anxiety, and stress). A confirmatory factor analysis on the raw data from the DASS-21 confirmed that a three-factor solution, χ^2^(186) = 886.757, CFI = 0.916, AIC = 36764.654, RMSEA = 0.067, 90% CI (0.062, 0.071), had a significantly better fit, Δχ^2^(3) = 673, *p* < .001, than a reduced one-factor solution, χ^2^(189) = 1559.755, CFI = 0.836, AIC = 37431.652, RMSEA = 0.093, 90% CI (0.088, 0.097).

All models were estimated hierarchically, testing first the main effects of empathy and emotion regulation on distress, and then the moderating effect of emotion regulation, via its interaction with empathy. All data were analysed in R 3.2.2 (R Core Team 2015), using packages fitdistrplus (Delignette-Muller and Dutang 2015), Hmisc (Harrell et al. 2015), psych (Revelle 2016), lavaan (Rosseel 2012), betareg (Cribari-Neto and Zeileis 2010), and lmtest (Zeileis and Hothorn 2002). As beta models use a logit link, estimates are presented as odds ratios. To reduce multicollinearity, continuous predictors were standardised prior to analysis. Wald *z*-tests were used to compare the equality of slope parameters across the depression, anxiety, and stress models.

## Results

### Main effects

Descriptive statistics and inter-correlations for the study variables are in Table 1. People with higher cognitive empathy stated they were more likely to reappraise, *rs* = .31, *p* < .001, and less likely to suppress, *rs* = − .08, *p* < .05, emotions. Higher trait cognitive empathy was associated with less depression, *rs* = − .16, *p* < .001, anxiety, *rs* = − .08, *p* < .05, and stress, *rs* = − .08, *p* < .05. People with higher affective empathy reported they were less likely to suppress emotions, *rs* = − .18, *p* < .001. Higher trait affective empathy was associated with greater anxiety, *rs* = .11, *p* < .01, and stress, *rs* = .18, *p* < .001. A greater tendency to reappraise emotion was linked to less depression, *rs* = − .28, *p* < .001, anxiety, *rs* = − .10, *p* < .01, and stress, *rs* = − .23, *p* < .001, while a greater tendency to suppress emotions was associated with increased depression, *rs* = .21, *p* < .001, anxiety, *rs* = .18, *p* < .001, and stress, *rs* = .11, *p* < .01.

**Table 1:** Descriptive statistics and inter-correlations of study variables

	1	2	3	4	5	6	7	8	9	10	11	12
1. Gender	–											
2. Age	0.02	–										
3. Nationality	−0.03	0.30***	–									
4. Postgraduate	0.01	0.58***	0.22***	–								
5. Relationship	0.13***	0.10*	−0.13***	0.08*	–							
6. Cognitive empathy	0.14***	0.04	−0.02	0.00	0.08*	–						
7. Affective empathy	0.27***	−0.04	−0.12***	−0.04	0.06^†^	0.26***	–					
8. Cognitive reappraisal	0.02	0.09**	0.15***	0.06^†^	−0.05	0.31***	−0.02	–				
9. Expressive suppression	−0.23***	−0.05	0.10**	−0.02	−0.20***	−0.08*	−0.18***	0.03	–			
10. Depression	−0.02	−0.11**	−0.03	−0.11**	−0.09*	−0.16***	0.05	−0.28***	0.21***	–		
11. Anxiety	−0.02	−0.07*	0.10**	−0.06^†^	−0.09**	−0.08*	0.11**	−0.10**	0.18***	0.60***	–	
12. Stress	0.06^†^	0.00	−0.01	−0.05	−0.03	−0.08*	0.18***	−0.23***	0.11**	0.68***	0.67***	–
Range	0–1	18–66	0–1	0–1	0–1	21–76	15–48	6–42	4–28	0–21	0–19	0–21
M	0.71	22.27	0.37	0.34	0.47	57.14	33.75	28.87	15.47	4.68	4.35	6.38
SD	0.46	5.05	0.48	0.47	0.5	8.28	5.51	6.39	4.96	4.49	4.04	4.39
Median	1	21	0	0	0	57	34	30	16	3	3	6
IQR	1	4	1	1	1	9	7	8	7	6	5	6
Skew (*z*-score)	−10.81	39.35	6.16	7.88	1.63	−5.14	−2.72	−7.48	−1.39	15.71	14.73	7.76
Kurtosis (*z*-score)	−6.98	99.71	−10.31	−9.29	−11.80	5.83	0.74	3.05	−2.78	7.81	6.46	−0.80

*N* = 844. Correlations represent Spearman’s rho (*rs*), rank-biseral (*r*_rb_), or phi (*r*_Φ_) coefficients

^†^*p* < .10; *p < .05.; **p < .01; ***p < .001

The results of the hierarchical beta regressions are in Table 2. Greater cognitive empathy predicted less depression, OR = 0.89, 95% CI (0.82, 0.96), *p* = .004, anxiety, OR = 0.91, 95% CI (0.84, 0.98), *p* = .011, and stress, OR = 0.92, 95% CI (0.86, 0.99), *p* = .025. In addition, cognitive empathy had a significant quadratic effect on stress, OR = 1.06, 95% CI (1.02, 1.10), *p* = .003; as the level of cognitive empathy increased, reported stress decreased until values of cognitive empathy reached > ~ *M* + 0.75 *SD*, where the relationship with stress became positive. Greater affective empathy predicted greater depression, OR = 1.15, 95% CI (1.07, 1.25), *p* < .001, anxiety, OR = 1.25, 95% CI (1.16, 1.34), *p* < .001, and stress, OR = 1.30, 95% CI (1.21, 1.40), *p* < .001, and had a significantly stronger effect on stress than depression, *z* = 2.24, *p* = .025. A greater tendency to reappraise emotions predicted less depression, OR = 0.75, 95% CI (0.69, 0.80), *p* < .001, anxiety, OR = 0.92, 95% CI (0.85, 0.98), *p* = .017, and stress, OR = 0.82, 95% CI (0.77, 0.88), *p* < .001. Cognitive reappraisal had significantly greater beneficial effects on reported depression, *z* = − 3.91, *p* < .001, and stress, *z* = − 2.19, *p* = .029, than anxiety. A higher tendency to suppress emotions predicted more depression, OR = 1.26, 95% CI (1.17, 1.35), *p* < .001, anxiety, OR = 1.19, 95% CI (1.11, 1.28), *p* < .001, and stress, OR = 1.15, 95% CI (1.08, 1.24), *p* < .001.

**Table 2:** Hierarchical beta regression models

Step 1	Depression (1)				Anxiety (2)				Stress (3)				Wald *z*-test
	OR	95% CI LO	95% CI HI	*p*	OR	95% CI LO	95% CI HI	*p*	OR	95% CI LO	95% CI HI	*p*	Difference
	Pseudo *R*^2^ = 0.13, logLik = 552.4, *p* < .001				Pseudo *R*^2^ = 0.09, logLik = 627.8, *p* < .001				Pseudo *R*^2^ = 0.10, logLik = 298.4, *p* < .001				
Intercept	0.32	0.22	0.45	.000	0.34	0.24	0.48	.000	0.35	0.25	0.49	.000	Null
Gender (1 = woman)	1.02	0.87	1.21	.783	0.96	0.82	1.13	.625	1.16	1.00	1.35	.058	3 ≥ 2
Age	1.00	0.99	1.02	.825	0.98	0.97	1.00	.038	1.00	0.99	1.02	.806	2 ≥ 3
International (1 = yes)	1.03	0.88	1.20	.742	1.38	1.19	1.60	.000	1.04	0.90	1.20	.598	2 > 1 and 3
Postgraduate (1 = yes)	0.78	0.66	0.92	.003	0.87	0.74	1.02	.089	0.90	0.78	1.05	.190	Null
Relationship (1 = yes)	0.90	0.77	1.04	.139	0.89	0.78	1.03	.124	0.91	0.79	1.04	.154	Null
Cognitive empathy (CE)	0.89	0.82	0.96	.004	0.91	0.84	0.98	.011	0.92	0.86	0.99	.025	Null
Affective empathy (AE)	1.15	1.07	1.25	.000	1.25	1.16	1.34	.000	1.30	1.21	1.40	.000	3 > 1
CE squared (CE^2^)	1.01	0.97	1.05	.722	1.03	0.99	1.07	.209	1.06	1.02	1.10	.003	3 ≥ 1
AE squared (AE^2^)	1.00	0.95	1.05	.854	1.02	0.97	1.07	.408	1.01	0.96	1.06	.655	Null
Cognitive reappraisal	0.75	0.69	0.80	.000	0.92	0.85	0.98	.017	0.82	0.77	0.88	.000	1 ≥ 3 > 2
Expressive suppression	1.26	1.17	1.35	.000	1.19	1.11	1.28	.000	1.15	1.08	1.24	.000	Null

Step 2	Pseudo *R*^2^ = 0.13, logLik = 559.7, Δχ^2^(8) = 14.61, *p* = .067				Pseudo *R*^2^ = 0.12, logLik = 644.5, Δχ^2^(8) = 33.47, *p* = .000				Pseudo *R*^2^ = 0.11, logLik = 305.7, Δχ^2^(8) = 14.72, *p* = .065				
CE × reappraisal	1.05	0.98	1.12	.200	0.97	0.90	1.03	.310	0.97	0.91	1.04	.432	Null
CE × suppression	1.03	0.95	1.11	.481	1.14	1.06	1.22	.000	1.10	1.03	1.18	.006	2 > 1
AE × reappraisal	0.93	0.86	0.99	.033	0.92	0.86	0.98	.011	0.96	0.90	1.03	.282	Null
AE × suppression	0.95	0.89	1.02	.162	0.98	0.92	1.05	.573	0.91	0.86	0.98	.008	Null
CE^2^ × reappraisal	1.00	0.96	1.04	.984	0.96	0.92	1.00	.053	1.01	0.97	1.05	.545	2 ≥ 3
CE^2^ × suppression	1.00	0.96	1.04	.919	1.06	1.02	1.10	.003	1.02	0.98	1.06	.352	2 > 1
AE^2^ × reappraisal	0.97	0.92	1.01	.115	0.97	0.93	1.01	.109	1.00	0.96	1.04	.971	Null
AE^2^ × suppression	0.95	0.91	0.99	.029	0.96	0.92	1.00	.066	0.99	0.95	1.04	.801	Null

*N* = 844. Continuous predictors standardised prior to analysis. Odds ratios and CIs calculated by exponentiation of log estimates, inferential tests conducted on the log scale. Wald test comparisons: > estimated effect is larger at *p* < .05; ≥ estimated effect is larger at *p* < .10

### Moderation effects

Individual differences in reappraisal significantly moderated the effect of affective empathy on depression, OR = 0.93, 95% CI (0.86, 0.99), *p* = .033, and anxiety, OR = 0.92, 95% CI (0.86, 0.98), *p* = .011. Differences in suppression significantly moderated the effect of cognitive empathy on anxiety, OR = 1.14, 95% CI (1.06, 1.22), *p* < .001, and stress, OR = 1.10, 95% CI (1.03, 1.18), *p* = .006, and the effect of affective empathy on stress, OR = 0.91, 95% CI (0.86, 0.98), *p* = .008. Finally, suppression significantly moderated quadratic effects of cognitive empathy on anxiety, 1.06, 95% CI (1.02, 1.10), *p* = .003, and affective empathy on depression, 0.95, 95% CI (0.91, 0.99), *p* = .029. Suppression had a significantly greater moderating effect on the linear, *z* = 2.19, *p* = .043, and quadratic, *z* = 1.99, *p* = .047, effect of cognitive empathy on anxiety than depression.

**Fig. 1:**
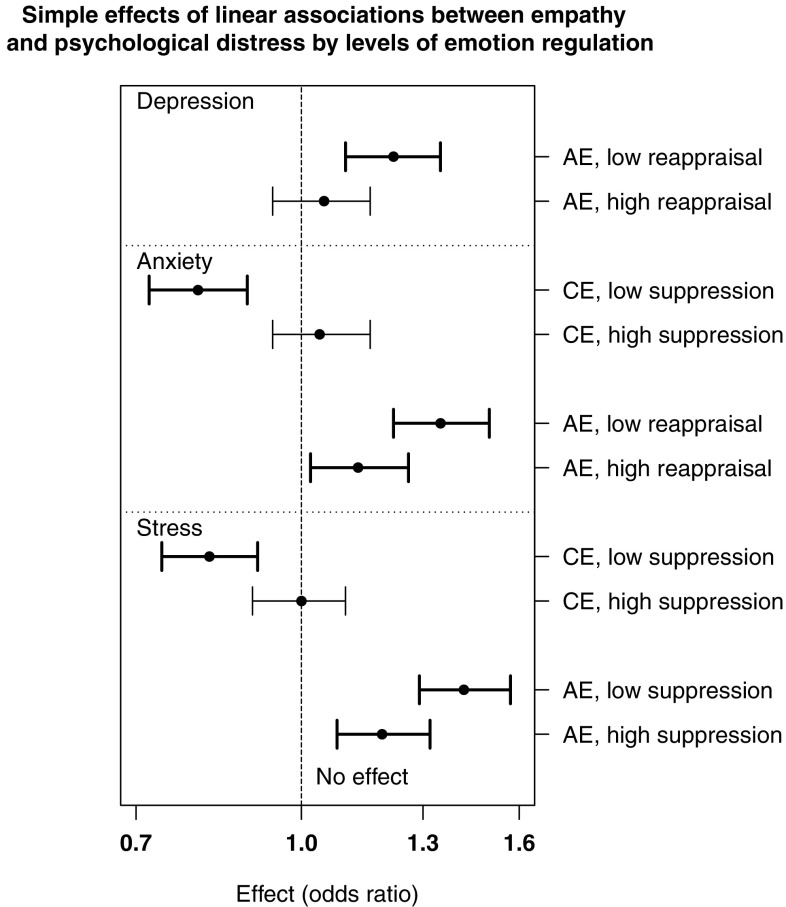
Simple effects (odds ratios) of significant linear interactions. Low values of the moderator represent − 1 *SD*, high values represent + 1 *SD*. Error bars represent 95% CIs. Bold bars are significant at *p* < .05. X-axis is on the log scale. *CE* cognitive empathy, *AE* affective empathy

**Fig. 2:**
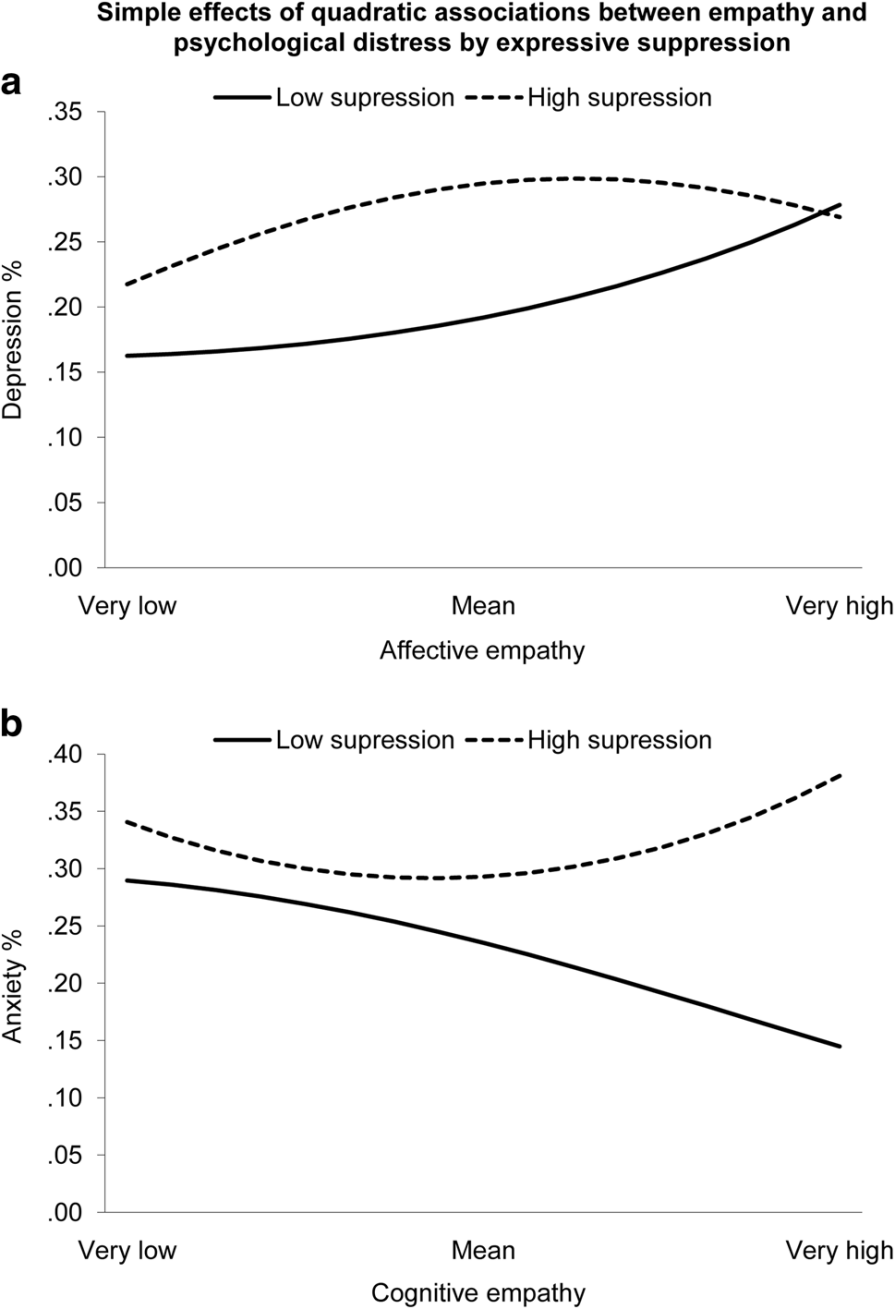
Simple effects of significant quadratic interactions. Low values of the moderator represent − 1 *SD*, high values represent + 1 *SD*. Slopes graphed between very low (− 2 *SD*) and very high (+ 2 *SD*) levels of the predictor variable

The effects of empathy on the three outcome variables at different levels of the moderators are presented in Figs. 1 and 2. Considering first the linear effects, affective empathy significantly predicted greater depression when the tendency to reappraise emotions was low (− 1 *SD*), OR = 1.22, 95% CI (1.10, 1.35), *p* < .001, but not when it was high (+ 1 *SD*), OR = 1.05, 95% CI (0.94, 1.16), *p* = .416. Affective empathy had a significantly stronger effect on anxiety when reappraisal was low, OR = 1.35, 95% CI (1.22, 1.50), *p* < .001, than when it was high, OR = 1.13, 95% CI (1.02, 1.26), *p* = .016. Cognitive empathy significantly predicted less anxiety when the tendency to suppress emotions was low, OR = 0.80, 95% CI (0.72, 0.89), *p* < .001, but not when suppression was high, OR = 1.04, 95% CI (0.94, 1.16), *p* = .401. Cognitive empathy also predicted less stress when suppression was low, OR = 0.82, 95% CI (0.74, 0.91), *p* < .001, not when suppression was high, OR = 1.00, 95% CI (0.90, 1.10), *p* = .924. Finally, affective empathy had a significantly stronger effect on stress when suppression was low, OR = 1.42, 95% CI (1.29, 1.57), *p* < .001, than high, OR = 1.19, 95% CI (1.08, 1.32), *p* < .001.

For the quadratic effects (Fig. 2), affective empathy did not have a significant quadratic relationship with depression when suppression was low, OR = 1.04, 95% CI (0.97, 1.11), *p* = .305, but began approaching significance when suppression was high, OR = 0.94, 95% CI (0.87, 1.00), *p* = .060. Cognitive empathy did not have a quadratic effect on anxiety when suppression was low, OR = 0.96, 95% CI (0.90, 1.03), *p* = .238, but had a significant quadratic effect when suppression was high, OR = 1.08, 95% CI (1.02, 1.14), *p* = .006.

## Discussion

This paper explored the theoretical model that the effects of individual differences in empathic abilities on common forms of affective distress may be moderated by differences in emotion regulation. First, consistent with prediction (1), cognitive empathy was negatively related to levels of depression, anxiety, and stress. This finding adds to prior research which indicates that, on average, greater levels of cognitive empathy are linearly associated with reduced depression (e.g., Cusi et al. 2011; Schreiter et al. 2013) and other positive socioemotional outcomes, such as sensitivity to injustice for others (Decety and Yoder 2015). Second, and consistent with prediction (2), affective empathy was positively related to all three forms of affective distress; a finding that supports previous work suggesting that empathic distress, from empathically experiencing the negative emotions of others, is often linked to reduced psychological wellbeing (e.g., Kahn et al. 2017; Schreiter et al. 2013). The independent opposing effects of cognitive and affective empathy on affective distress are congruent with a wider literature emphasising their distinctiveness (e.g., Bird and Viding 2014). The results are also consistent with a recent investigation demonstrating that cognitive empathy negatively predicted behavioural apathy, while affective empathy was a positive predictor (Lockwood et al. 2017); motivational apathy has close links with the symptoms of affective distress, and states of depression in particular.

Of note, there was only marginal support for prediction (3), that there may be an optimal or normative level of empathy, neither too low nor too high, in predicting lower levels of distress. Replicating the study by Tully et al. (2016), no significant quadratic effects of affective empathy on distress were found. However, the quadratic relationship between cognitive empathy and depression observed in Tully et al. (2016) failed to replicate here. Instead, a significant quadratic effect of cognitive empathy on stress was observed, and the size of this effect was marginally significantly larger than the quadratic effect of cognitive empathy on depression, suggesting that the result previously published may have been driven by shared variance between the two constructs. The effect of affective empathy on stress was also significantly larger than its effect on depression, suggesting that empathy may have a stronger effect on stress-related outcomes, which are more common, than depression (see e.g., Manczak et al. 2016).

On the basis of past work (e.g., John and Gross 2004), predictions (4) and (5) were that higher levels of cognitive reappraisal would be negatively associated, and higher levels of expressive suppression would be positively associated, with increased distress. The results were consistent with these expectations. The ability to reappraise one’s emotional states has been linked to adaptive psychological outcomes in survey-based (e.g., John and Gross 2004), experimental (e.g., Troy et al. 2010), and experiential sampling (e.g., Brans et al. 2013) studies. While similar levels of evidence have tied the suppression of emotion with negative consequences (e.g., Brans et al. 2013; Butler et al. 2003; John and Gross 2004). A novel finding was that the protective effect of cognitive reappraisal was marginally significantly larger for depressive symptoms than stress, which was significantly larger than its effects on anxiety. Thus, the current results reinforce and extend upon what is known about the association between emotion regulation and psychological wellbeing, while also suggesting that certain traits (i.e., the capacity for cognitive reappraisal) may be more beneficial for certain types of psychological outcomes (i.e., depression) than others (i.e., stress and anxiety).

The present results provide new evidence that individual differences in the emotion regulation techniques of reappraisal and suppression critically moderate some of the associations between empathy and distress (cf., Tully et al. 2016). Some support was found for prediction (6); for depression and anxiety, the detrimental effects of increased affective empathy were offset when people reported being more effective at cognitive reappraisal. This reinforces the role of cognitive reappraisal as a protective factor for empathy-induced distress outcomes and is consistent with work supporting reappraisal as an adaptive strategy for managing negative emotion (e.g., Brans et al. 2013; John and Gross 2004; Troy et al. 2010). There was no evidence that reappraisal significantly enhanced the already positive effects of cognitive empathy. Similar levels of support were documented for prediction (7); for anxiety and stress, the benefit of greater levels of cognitive empathy was absent in those who reported suppressing their emotions, and this moderating effect was significantly larger on anxiety than depression. For anxiety, suppression was a significant moderator of the quadratic effect of cognitive empathy, suggesting that higher levels of suppression were particularly detrimental to those with levels of cognitive empathy higher than or equal to the mean. These findings support a wider literature that indicates that, on average, suppressing emotion has negative psychological ramifications (e.g., Brans et al. 2013; Butler et al. 2003; John and Gross 2004). There was no evidence that suppression potentiated the positive effects of affective empathy on affective distress outcomes.

An unexpected, and unpredicted, finding was that higher levels of suppression appeared to have a potentially beneficial effect on levels of depression and stress for those higher in affective empathy. This challenges the conviction that suppression is always bad, especially in those with heightened affectivity (see e.g., Rogier et al. 2017). Indeed, looking at the quadratic interaction in Fig. 2, expressive suppression only began to have a positive moderating effect on the link between affective empathy and depression at levels of affective empathy above the mean, where contagious affectivity is at its highest. Recent studies have suggested that negative outcomes typically associated with expressive suppression may be moderated by gender (Rogier et al. 2017) or culture (Soto et al. 2011), and there is a need for more research exploring under what conditions suppression could be beneficial.

Overall, the results of the moderation analyses appear inconsistent with Tully et al. (2016), but this study uses nearly three times the sample size used in that study, improved statistical techniques (e.g., modelling the predictors simultaneously), and a mixed sample (rather than an exclusive reliance on psychology students), which could explain the discrepant findings. It also extends the evidence base to consider common forms of affective distress beyond depression, with effects that differ significantly in magnitude. The findings are consistent with complementary work that has demonstrated a significant moderating effect of cognitive reappraisal between affective empathy and prosocial tendencies (Lockwood et al. 2014) but suggest that the moderating effect may operate differently depending on the outcome(s) of interest.

These findings have implications for emotional interventions designed to reduce or buffer against common forms of affective distress, suggesting that the targets of such interventions may differ depending on people’s empathic traits. For people with a higher propensity for affective empathy, this evidence suggests that fostering reappraisal is a particularly important quality. Conversely, there was indicative evidence that levels of suppression were not necessarily always bad for those exhibiting high levels of affective empathy (see e.g., Rogier et al. 2017). On the other hand, for those high in cognitive empathy, higher levels of expressive suppression appear to nullify otherwise beneficial effects of cognitive empathy on anxiety and stress. Accordingly, training in emotion regulation strategies could be targeted based on dispositional levels of empathy. Interventions to enhance reappraisal in the laboratory have been shown to be successful (e.g., Miu and Crişan 2011), and to be expandable to applied settings (e.g., Halperin et al. 2013). Similarly, techniques are available to up- (e.g., Schneider et al. 2013) or down- (e.g., Dick et al. 2014) regulate expressive suppression.

Some limitations of the current study should be noted. While the sample size is large, it is still a—albeit mixed—student sample, potentially limiting generalisability to non-student populations. Second, the work relies on self-report data, and differences between objective and self-report data in the association between empathy and depression have been noted (Schreiter et al. 2013), yet the work makes a unique contribution with a sample size often unachieved with objective methods. Third, the questionnaires were measured on different response scales, potentially artificially reducing variance on the QCAE and DASS-21 (which used 4-point scales) relative to the ERQ (which used a 7-point scale). Although the multi-item nature of the questionnaires should help to reduce the impact of this limitation. Fourth, no alpha correction was applied, as procedures such as the Bonferroni correction can render analyses extremely conservative as a function of the number of tests reported (Perneger 1998). Accordingly, the findings reported, and particularly the Wald test comparisons, should be considered exploratory, warranting further confirmation in future studies. Finally, the work is cross-sectional, limiting claims of directionality. However, the model is based on a strong theoretical precedent: individual differences in empathy produce different emotional experience(s), which are the target of emotion regulation strategies, and contribute to affective distress as the product of emotional disturbance (Powell et al. 2013).

In conclusion, the current study is the first to provide evidence supporting a moderating (i.e. offsetting) effect of individual differences in two, well-established emotion regulation techniques on the link between empathy and affective distress. The findings suggest that if a person is higher in affective empathy, reappraisal is a particularly good strategy to avoid distressing outcomes, and that suppression may also have some utility. If someone is higher in cognitive empathy, on the other hand, suppression is consistently a bad regulation strategy and should be discouraged, in order to maximise benefit to psychological wellbeing.

## Electronic supplementary material

Below is the link to the electronic supplementary material.

Supplementary material 1 (DOCX 1681 KB)

Supplementary material 2 (XLSX 55 KB)
