# Supplementary material for: Individual differences in emotion regulation moderate the associations between empathy and affective distress
Source: Motiv Emot. 2018 Mar 14;42(4):602–13. doi: 10.1007/s11031-018-9684-4 (PMC5982456; doi:10.1007/s11031-018-9684-4)
Supplement: Supplementary file 1 — Supplementary material 1 (DOCX 1681 KB) [file 11031_2018_9684_MOESM1_ESM.docx]

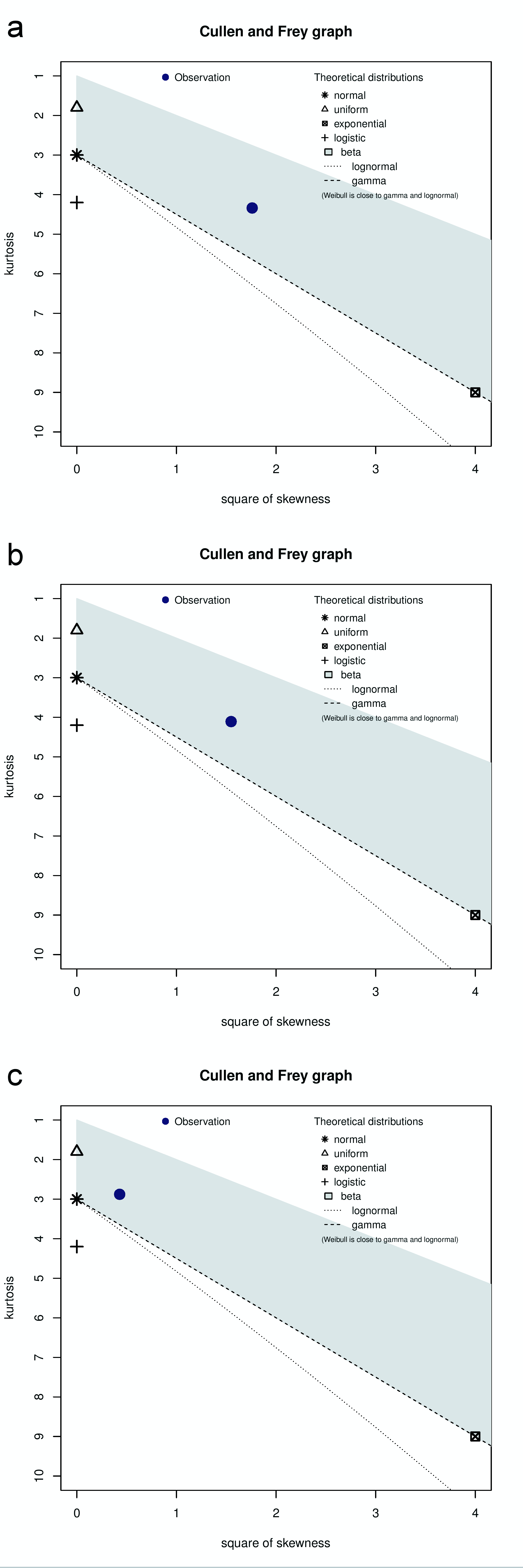


*Figure S1*. Cullen and Frey graphs (Cullen & Frey, 1999) illustrating the likely theoretical distribution of the data based on observed values of skewness and kurtosis for the depression (a), anxiety (b), and stress (c) outcomes.


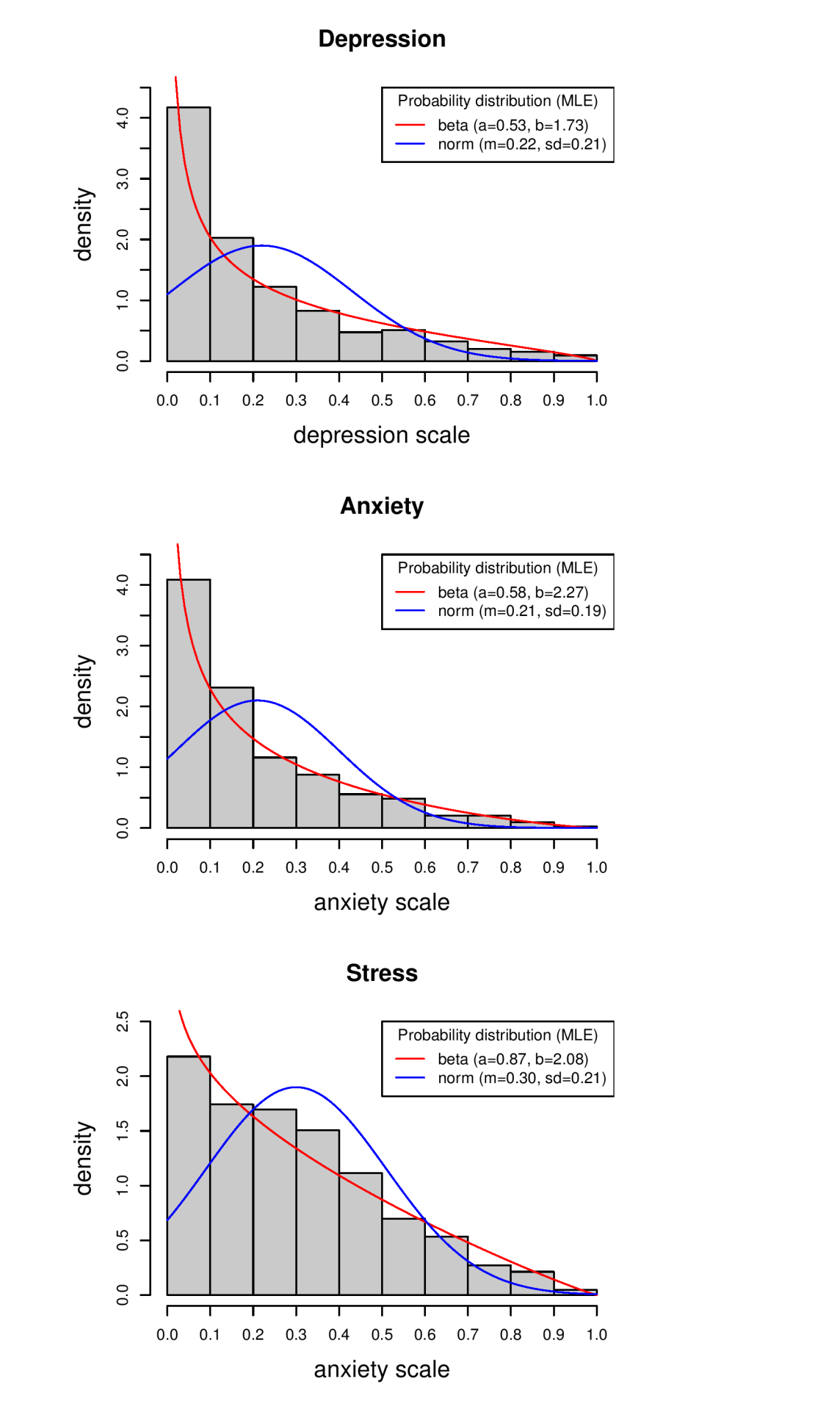


*Figure S2*. Density histograms illustrating the distributions of the primary outcome variables (depression, anxiety, and stress). Beta and normal probability distributions are overlaid, with optimal shape parameters determined by maximum likelihood estimation (MLE). The beta distribution represents the observed data better than the normal distribution.
